# Supplementary material for: Serpin-4 Negatively Regulates Prophenoloxidase Activation and Antimicrobial Peptide Synthesis in the Silkworm, Bombyx mori
Source: Int J Mol Sci. 2023 Dec 25;25(1):313. doi: 10.3390/ijms25010313 (PMC10778760; doi:10.3390/ijms25010313)
Supplement: Supplementary file 1 [file ijms-25-00313-s001.zip › ijms-2717028-supplementary.pdf]

Supplemental file S1 The comparisons of the amino acid sequences of MsHP1 vs BmHP1, MsHP5 vs BmSP2, MsHP6 vs BmHP6, and MsHP21 vs BmSP21.

MsHP1 vs **BmHP1** (**74%** identities of amino acid residues)

```
B.mori_HP1_BAG70409.1     MVRVLLLLLTVAFVKADGDFSELTDPAWRDVIENGGLHIG   40
M.sexta_HP1_AAB94557.1    MIFVFLLLL.VSVCSEKGDYSELQHPAWEDIIEEGGMHIG   39
Consensus                 m  v llll v      gd sel  paw d ie gg hig

B.mori_HP1_BAG70409.1     SGRTKRFIQLNTNQANIPYQSCTLPNGKAGRCRQLRHCIQ   80
M.sexta_HP1_AAB94557.1    GGRSKRFIQLNPNQGNVAYQACTLPSGKQGHCRHLRFCIQ   79
Consensus                  gr krfiqln nq n  yq ctlp gk g cr lr ciq

B.mori_HP1_BAG70409.1     EDFKKDYLVFMDYVCVIERSSIGVCCPENEVKEGIEALAG  120
M.sexta_HP1_AAB94557.1    EDFKQDFVKFMNYVCVIQGQSMGVCCPEDTTVGGPEGLAG  119
Consensus                 edfk d   fm yvcvi   s gvccpe     g e lag

B.mori_HP1_BAG70409.1     DLPATAPKNEDDEILLKINRAENRGCGLSTRAQGRITGSR  160
M.sexta_HP1_AAB94557.1    DLPATAPQEGEDETLLKINQAQNRGCGLSTRAQGRVFGSR  159
Consensus                 dlpatap    de llkin a nrgcglstraqgr  gsr

B.mori_HP1_BAG70409.1     PANPREWPWMASITPYGFEQYCGGVLITDRHVLTAAHCTR  200
M.sexta_HP1_AAB94557.1    PANPREWPWMASITPEGFEQYCGGVLITDRHVLTAAHCTR  199
Consensus                 panprewpwmasitp gfeqycggvlitdrhvltaahctr

B.mori_HP1_BAG70409.1     RWDADELYVRLGEYDLQRTNDSRSYNFKVVEKIQHPNFEL  240
M.sexta_HP1_AAB94557.1    RWEANELYVRLGEYDFKRTNDTRSYNFRVVEKVQHVDFEI  239
Consensus                 rw a elyvrlgeyd  rtnd rsynf vvek qh  fe

B.mori_HP1_BAG70409.1     SSYHNDIAILKLHRPAVFNTYVWPICLPPADLDLTNEIAT  280
M.sexta_HP1_AAB94557.1    SNYHHDIAILKLDKPAIFNTYVWPICLPPPGLSIENETVT  279
Consensus                 s yh diailkl  pa fntyvwpiclpp  l   ne  t

B.mori_HP1_BAG70409.1     VIGWGTQWYGGPHSNVLMEVSVPVWDHQKCVDAFVDSVFT  320
M.sexta_HP1_AAB94557.1    VIGWGTQWYGGPHSHVLMEVSFPIWTHQNCIEVHTNSIFD  319
Consensus                 vigwgtqwyggphs vlmevs p w hq c      s f

B.mori_HP1_BAG70409.1     ETVCAGGLEGGKDACQGDSGGPLMYQMSSGRWAVVGVVSW  360
M.sexta_HP1_AAB94557.1    ESICAGGHEGGRDACQGDSGGPLMYQMPSGRWAVVGIVSW  359
Consensus                 e  cagg egg dacqgdsggplmyqm sgrwavvg vsw

B.mori_HP1_BAG70409.1     GLRCGEPNHPGLYARVDKYLDWILLNSR              388
M.sexta_HP1_AAB94557.1    GVRCGEPNHPGIYTRVDKYIGWIMENAR              387
Consensus                 g rcgepnhpg y rvdky  wi  n r
```

MsHP5 vs **BmSP2** (52% identities of amino acid residues)

```
M.sexta_HP5_QIG55613.1         MFNVNVFVYLFLLGLLGVGSLFAGDGCYVEENQGTCVVLS     40
B.mori_SP2_XP_037876437.1      MLQT...FFLVVTSILQIQSLEFGESCFANDEPGTCIALS     37
Consensus                      m        l     l   sl  g  c      gtc  ls

M.sexta_HP5_QIG55613.1         DCQHLINEIHRAGKPMPIHIRNKLQMLGCGFESDKPMVCC     80
B.mori_SP2_XP_037876437.1      ECKPVLSYIEKFGNQIPNNLLQKFQDLTCGYDHSDPLVCC     77
Consensus                       c      i   g   p     k q l cg     p vcc

M.sexta_HP5_QIG55613.1         VPPSNFDAINSWTPEIHGSSLSGVPNRGKPTQTDDNVVWG    120
B.mori_SP2_XP_037876437.1      TTSQHIAGYFNNFGNQNGG..TSVPKRPKPVPTSNSGNWG    115
Consensus                                       g     vp r kp  t     wg

M.sexta_HP5_QIG55613.1         SDYSN....SNIPPKDDTDILGHEHDKLGQFTPSDRVTTT    156
B.mori_SP2_XP_037876437.1      SQPSKEQTTSNRPTTADTDINKSGRD........DDFPDI    147
Consensus                      s  s     sn p   dtdi     d        d

M.sexta_HP5_QIG55613.1         ANHRNIGLLPTNCGSIESDRIIGGNRTRLFEMPWMVLLSY    196
B.mori_SP2_XP_037876437.1      RQNPNLSLLSSNCGTIESDRIFGGNRTRLFEMPWMVLLSY    187
Consensus                          n  ll  ncg iesdri ggnrtrlfempwmvllsy

M.sexta_HP5_QIG55613.1         QSGRRTRLDCGGTLINEWYVLTAAHCVTSLRSNLILTHVI    236
B.mori_SP2_XP_037876437.1      DSDRGTKLNCGGTLINEWYVLTAAHCVSFLGNRLTLKHVI    227
Consensus                       s r t l cggtlinewyvltaahcv  l   l l hvi

M.sexta_HP5_QIG55613.1         LGEHDVEHDPDCERSDGNKYCAPPIKTVTIEETIPHPRYN    276
B.mori_SP2_XP_037876437.1      LGEYDTRQDPDCERSEGKEYCAAGIITAEFDEVIPHTGYT    267
Consensus                      lge d   dpdcers g  yca  i t    e iph  y

M.sexta_HP5_QIG55613.1         SKTFADDIALLRLSEPADFNLDNMKPLCLPLTLQLQTENL    316
B.mori_SP2_XP_037876437.1      PQTLIDDIALIRLKEPADFDLDNIKAICLPITPELQREPL    307
Consensus                        t  ddial rl epadf ldn k  clp t  lq e l

M.sexta_HP5_QIG55613.1         VNINGIVAGWGVTEEGMESSVLLSVSLPILSKDECETAYK    356
B.mori_SP2_XP_037876437.1      VDNFGVVAGWGVTEEGLQSPVLLSVQLPILSKEKCLQDYS    347
Consensus                      v   g vagwgvteeg  s vllsv lpilsk  c   y

M.sexta_HP5_QIG55613.1         G.TVQLSDKQLCAGGVRDKDSCGGDSGGPLMYPGKLGPGG    395
B.mori_SP2_XP_037876437.1      QYSLKINDKQLCAGGLHDKDSCAGDSGGPLLYPGKVGSTG    387
Consensus                             dkqlcagg  dkdsc gdsggpl ypgk g  g

M.sexta_HP5_QIG55613.1         IKYIQRGIVSYGTKRCGVGGFPGVYTNVASYMDWILDNMH    435
B.mori_SP2_XP_037876437.1      VRYVQRGIVSFGSKRCGIS.LPGVYTNVAYYMDWILNNIR    426
Consensus                        y qrgivs g krcg    pgvytnva ymdwil n
```

MsHP6 vs **BmHP6** (65% identities of amino acid residues)

```
B.mori_HP6_XP_037868595.1    MRQYGPKHLIWILIISGILIKAEDVGDECTPNNQISEGIC    40
M.sexta_HP6_AAV91004.1       MWLMVNNIILCLLITN..SIIAENVGDECTPSSSTGDGTC    38
Consensus                    m           li     i ae vgdectp       g c

B.mori_HP6_XP_037868595.1    TLVNDCPQAVMAIKNKRFQPFQRCGFRGFQEIVCCPTTVD    80
M.sexta_HP6_AAV91004.1       TLVSDCPAAIRAIKNKRFHEFQRCGFDGFQEIVCCPLTTD    78
Consensus                    tlv dcp a  aiknkrf  fqrcgf gfqeivccp t d

B.mori_HP6_XP_037868595.1    KFGQTEKT.RTIKRIAERECDKIISSTVPPLDLYILGGEA   119
M.sexta_HP6_AAV91004.1       KFGATETSPKVAQRITDRECKTILAGTIPPLDLHILGGEE   118
Consensus                    kfg te        ri  rec  i    t ppldl ilgge

B.mori_HP6_XP_037868595.1    ASMGEFPYMVAVGFDRGNG.YEFDCGGSLLSNLYVLTAAH   158
M.sexta_HP6_AAV91004.1       ASLGEFPHMVALGFDNGGGEYRFDCGGSLISNYYVLTAAH   158
Consensus                    as gefp mva gfd g g y fdcggsl sn yvltaah

B.mori_HP6_XP_037868595.1    CVDTLDRIEPSLVRVGVIELGSNAWTEGTDYRIAQIITHP   198
M.sexta_HP6_AAV91004.1       CIDTADREPPSVVRAGVVNIGGPAWDDETDYRVAETILHP   198
Consensus                    c dt dr  ps vr gv    g  aw   tdyr a   i hp

B.mori_HP6_XP_037868595.1    NYTRREKYHDLALLRLEKAVAFSSNVNAVCLETSTEDPTV   238
M.sexta_HP6_AAV91004.1       NYTRREKYHDVALLRLDRPVQFSSTLNAVCLFSSNENPTS   238
Consensus                    nytrrekyhd allrl    v fss  navcl  s e pt

B.mori_HP6_XP_037868595.1    PLTITGWGKISNTRNAKSNILLKANVTAVKAEKCSESYLN   278
M.sexta_HP6_AAV91004.1       KLTITGWGRTSNTRDIKSSKLLKADVVVVPSDKCGESYTN   278
Consensus                     ltitgwg  sntr  ks  llka v  v   kc esy n

B.mori_HP6_XP_037868595.1    WRKLPKGISDSQICAGDPEGIRDTCQGDSGGPLQMWR..D   316
M.sexta_HP6_AAV91004.1       WRKLPHGISQEMMCAGDPKGVRDTCQGDSGGPLQLMEKDG   318
Consensus                    wrklp gis    cagdp g rdtcqgdsggplq

B.mori_HP6_XP_037868595.1    VYRLVGVTSFGRGCGSPVPGVYTRLSRYLDWIESVVWPNH   356
M.sexta_HP6_AAV91004.1       LYRLVGVTSFGRGCGSYVPGVYTRVSNYLGWIESIVWPN.   357
Consensus                     yrlvgvtsfgrgcgs vpgvytr s yl wies vwpn
```

MsHP21 vs **BmSP21** (56% identities of amino acid residues)

```
M.sexta_HP21_AAV91019.1       ...MLREVLLVALCIVVRAADENETCNMKNGEVGICKNIR    37
B.mori_SP21_NP_001243984.1    MDQSIYLLLMSVLFVCVHCEFEGEECKKGN.LLGVCTNIR    39
Consensus                             l   l   v    e e c   n   g c nir

M.sexta_HP21_AAV91019.1       NCPSALENLRKRIQPQLCGFDKSDPIVCCVESVTTPAPTQ    77
B.mori_SP21_NP_001243984.1    KCQSALNDIRNRKSPQICSFDNADPVVCCFDNSIS...SR    76
Consensus                      c sal   r r  pq c fd  dp vcc

M.sexta_HP21_AAV91019.1       PPIATTTKRPQVTTTTEYEPPLYEYETVDRQGSGCPPIDA   117
B.mori_SP21_NP_001243984.1    APLATTTRRTPSSTTTEYVPPSYDYQSNNGD.KKCEDVPA   115
Consensus                      p attt r    tttey pp y y         c    a

M.sexta_HP21_AAV91019.1       NLTSPKIGRKAWDKCLEYQEKLVYPCEKSFSLSLNDAMER   157
B.mori_SP21_NP_001243984.1    DLTSPKTGQKAWDKCIEYQEQLVYPCEKGVALTG..EISR   153
Consensus                      ltspk g kawdkc eyqe lvypcek   l       r

M.sexta_HP21_AAV91019.1       KVKCHNNADDLIIGGQNASRNEFPHMALLGYGEEPD.VQW   196
B.mori_SP21_NP_001243984.1    SKHCHHDADELIIGGTDAGVNEYPHMVLLGYGDDVANIQW   193
Consensus                        ch  ad liigg  a  ne phm llgyg      qw

M.sexta_HP21_AAV91019.1       LCGGTLISENFILTAGHCISSRDIN.LTYVYLGALARSEV   235
B.mori_SP21_NP_001243984.1    LCGGVLISERFVLTAGHCLSSREVGAVRYVYIGALARHET   233
Consensus                     lcgg lise f ltaghc ssr      yvy galar e

M.sexta_HP21_AAV91019.1       TDPSKQYRIKKIHKHPEFAPPVRYNDIALVELERNVPLDE   275
B.mori_SP21_NP_001243984.1    TNPSRRYAVIRAHRHPDYKPPSKYNDIALLELDRQVPLDQ   273
Consensus                     t ps  y     h hp   pp  yndial el r vpld

M.sexta_HP21_AAV91019.1       WLKPACLHMGDETADDRVWATGWGLTEYKASSGANILQKV   315
B.mori_SP21_NP_001243984.1    YTVPACLHTGDAVNDERASATGWGLTENRGST.SDVLQKV   312
Consensus                        paclh gd   d r  atgwglte   s     lqkv

M.sexta_HP21_AAV91019.1       VLNKFSTFECILQYPPHRLMSQGFDVNSQMCYGDRSQSKD   355
B.mori_SP21_NP_001243984.1    VLTKFTSAECSEKYPTNRNMKRGFDPRTQMCYGDRTLSRD   352
Consensus                     vl kf   ec   yp  r m  gfd   qmcygdr  s d

M.sexta_HP21_AAV91019.1       TCQGDSGGPLQIKHKKINCMWLIIGVTSFGKACGFIGEPG   395
B.mori_SP21_NP_001243984.1    TCQGDSGGPIQIKSKKMDCMYVVIGVTSFGRACGYAGEPG   392
Consensus                     tcqgdsggp qik kk  cm   igvtsfg acg  gepg

M.sexta_HP21_AAV91019.1       IYTKVSHYIPWIESVVW                          412
B.mori_SP21_NP_001243984.1    IYTRVSHYVPWIESVVW                          409
Consensus                     iyt vshy pwiesvvw
```
